# Clinical and subclinical features and MEFV mutation distribution in of FMF patients’ siblings

**DOI:** 10.1186/1546-0096-13-S1-P92

**Published:** 2015-09-28

**Authors:** Z Gunduz, B Sozeri, A Esen, A Pac Kısaarslan, H Kılıc, R Dusunsel, H Poyrazoglu, M Dundar, I Dursun

**Affiliations:** 1 Erciyes University Faculty of Medicine, Pediatric Rheumatology, Kayseri, Turkey; 2 Erciyes University, Faculty of Medicine, Department of Genetics, Kayseri, Turkey

## Objective

Familial Mediterranean fever (FMF) is an autosomal recessive disease characterized by recurrent attacks of fever and peritonitis, pleuritis, arthritis or an erysipelas-like skin disorder. The disease may present at any age, more than 80% of patients being symptomatic by the age of 20 yr. Its main long-term complication is amyloid A (AA) amyloidosis, a severe manifestation with poor prognosis. Mutations in the MEFV gene, on chromosome 16^th^, is encoding a protein named as pyrin. We aim to analyze clinical characteristics, subclinical inflammation, and carried MEFV mutation in siblings with FMF.

## Methods

We obtain FMF patients who were followed in Erciyes University Faculty of Medicine, department of pediatric rheumatology and their siblings. All children were evaluated with a questionnaire containing 21 questions which symptoms may be signs of FMF. All subjects were invstigated for laboratory disease features, genetic analysis of MEFV mutations.

## Results

The study included 53 pediatric patients and their 76 sibling total 129 children from 50 different families. In patients, the most frequent mutations were homozygous pM694V (60%), pM694V/pM680I (9.5%), pM694V/pV726A (3.6%) and heterozygous pM694V mutation (3.6%). Therefore, in siblings of the patients had mostly homozygous pM694V (12%), pE148Q/P369S and heterozygous pM694V mutation (35.5%) while 11 (14.5%) sibling had no mutation in MEFV gene. We also completed a questionnaire form included findings of FMF in all subject. We obtained the typical history of FMF (the presence of fever, recurrent typical attacks of FMF (including peritonitis, pleuritis and arthritis); and transient inflammatory response) in 9 of 76 siblings (12%) from the questionnaire. In addition, the presence of rare but important manifestations such as erysipelas like erythema and leg pain of FMF were determine in patients’ siblings. The exertional leg pain had found in 21 siblings (28%) who carried at least one mutation in the MEFV gene. Also we evaluated acute phase reactants (ESR, CRP, SAA and S100A protein) in all subjects. The siblings with homozygous mutation had elevated levels of SAA and S100A protein than others.

## Conclusion

Our findings showed that siblings with FMF had different clinical findings each other. In these children should be questioned in terms of FMF findings before screen mutation in MEFV. Fever, serositis symptoms and musculoskeletal symptoms in children, especially presence of homozygous mutation, even if there has no typical attacks, we believe that the colchicine treatment should be considered.

